# A Comparison between Video and Still Imagery as a Methodology to Determine Southern Hairy-Nosed Wombat (*Lasiorhinus latifrons*) Burrow Occupancy Rates

**DOI:** 10.3390/ani8110186

**Published:** 2018-10-23

**Authors:** Michael J. Swinbourne, David A. Taggart, Bertram Ostendorf

**Affiliations:** School of Biological Science, University of Adelaide, L3 Oliphant Bldg, North Terrace, Adelaide, SA 5005, Australia; david.taggart@adelaide.edu.au (D.A.T.); bertram.ostendorf@adelaide.edu.au (B.O.)

**Keywords:** wombats, warrens, species abundance, wildlife cameras

## Abstract

**Simple Summary:**

While many people have great affection for southern hairy-nosed wombats, they are also considered by others to be an agricultural pest, because of the damage they can cause to farmland and agricultural infrastructure. Therefore, we need to have a good understanding of how many wombats there might be and how the population is changing, if we are to make properly informed decisions on how to best manage them. Unfortunately, because wombats are nocturnal and live underground, and because they use a number of different burrows throughout their home ranges, counting them can be difficult. We used motion-activated cameras to record how often wombats use each burrow in order to develop a reliable method of counting wombats that we can apply at the broad scale. We found that, on average, there are around 0.43 wombats for each active burrow. The use of video cameras to record this information provided a much simpler and less invasive means of researching wombat behaviour than methods such as trapping. However, video cameras do have limitations that need to be considered, and researchers need to fully understand their capabilities and limitations before employing them in the field.

**Abstract:**

Broad-scale abundance estimates of the southern hairy-nosed wombat population use a proxy measure based on counting the number of active burrows, which is multiplied by an index of ‘wombats/active burrow’. However, the extant indices were calculated in the 1980s, prior to the use of calicivirus to control rabbits, and used invasive monitoring methods which may have affected the results. We hypothesise that the use of video might provide a logistically simple, non-invasive means of calculating updated indices. To this end, motion-activated, infra-red still and video cameras were placed at various distances outside active wombat burrows in the South Australian Murraylands and Eyre Peninsula regions. The captured imagery was inspected to determine how often the burrow was occupied by one or more wombats, and how effective the cameras were at detecting wombat activity. Video data was clearly superior to the still imagery, with more than twice as many burrow occupancies being positively identified (still: 43%). The indices of wombats/active burrow calculated based on video imagery were: Murraylands: 0.43, Eyre Peninsula: 0.42. 1948 false positive videos were recorded, of which 1674 (86%) occurred between noon and sunset.

## 1. Introduction

**Figure 1:**
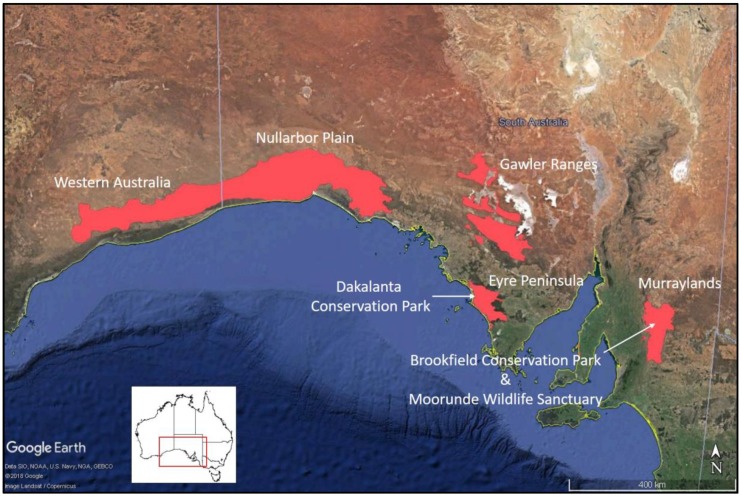
Distribution of southern hairy-nosed wombats as at the most recent assessments in 2016–2018 [1].

Southern hairy-nosed wombats (*Lasiorhinus latifrons*) inhabit a fragmented distribution across the semi-arid regions of south-central South Australia and the south-eastern corner of Western Australia (Figure 1). While recent studies have been able to map the species-wide distribution of southern hairy-nosed wombats using very high-resolution satellite imagery [1], determining how many wombats there might be is more problematic [2,3,4]. Wombat abundance at the broad-scale is estimated by using a proxy measure of counting the number of active burrows (burrows which are currently being used by wombats), which is then multiplied by an index of the number of wombats/active burrow. However, there are several problems with this approach.

Indices of the number of wombats/active burrow were first calculated in the 1980s, based on mark-recapture studies using cage traps installed at burrow entrances, which were conducted near Fowlers Bay on the west coast of South Australia [5] and near Blanchetown in the Murraylands [6]. The number of individual wombats caught in the traps were counted, and the result was divided by the number of active burrows being surveyed. While hairy-nosed wombats generally have a preferred warren in which they reside for the majority of the time, they also use up to ten different warrens throughout their home range [7]. As a result, indices of the number of wombats/active burrow based on how many individual wombats there may be in a warren could potentially over-estimate the index by counting not only the wombats which regularly reside in the warren, but also wombats who were visiting from nearby warrens. The use of invasive approaches such as cage traps and mark-recapture surveys also have the potential to skew the results, as wombats will adopt measures such as remaining in their burrows for up to 10 days to avoid the traps [8].

We hypothesise that, rather than attempting to identify the number of wombats in an area and then relating that to the number of active burrows (i.e., the object of the study being the number of wombats), a more accurate assessment of the number of wombats/active burrow can be obtained by determining the proportion of active burrows which are being simultaneously occupied by wombats (i.e., the object of the study is the burrow occupancy rate). To achieve this, a reliable method of determining when wombats enter and exit each burrow, and how many wombats are using the burrow at the same time, is required; one which does so non-invasively to minimise any changes in the animals’ behaviour.

Motion-activated infra-red cameras (camera traps/wildlife trail cameras) offer a relatively cheap and logistically simple means of monitoring wildlife behaviour [9]. The use of motion-activated cameras for wildlife research in Australia has increased significantly over the past decade, with an exponential increase in the number of published papers since 2010 describing the use of camera traps [10]. While much of the increase in use of motion-activated cameras can be attributed to improvements in the capability and reductions in the cost of the cameras, improvements in both the capacity and cost of data storage has also been important [11]. Wildlife trail cameras can now capture and store tens of thousands of high-resolution day or night-time images, meaning that they can be left in place longer and can collect more data than earlier camera models. The growth in data storage capacity has also facilitated the greater use of video to analyse wildlife behaviour. This has allowed for a greater range and a more accurate interpretation of animal behaviour than would otherwise be possible using still imagery alone [12]. However, while still imagery has been used on a number of occasions for wombat research [4], there has been no published research on the use of video to monitor wombat behaviour in the wild.

The aims of this study were to compare the effectiveness of video and still imagery for determining how often the active burrows of southern hairy nosed wombats are utilised, and to use video and still imagery to calculate an index of the number of wombats/active burrow based on burrow occupancy rates.

## 2. Materials and Methods

This study was conducted in two phases. Phase one was conducted in the Murraylands region of South Australia in late spring/early summer 2017. The results from Phase one were analysed and used to refine the camera settings for the Phase two, which was conducted on the Eyre Peninsula during autumn 2018.

### 2.1. Phase 1: Murraylands Region

**Figure 2:**
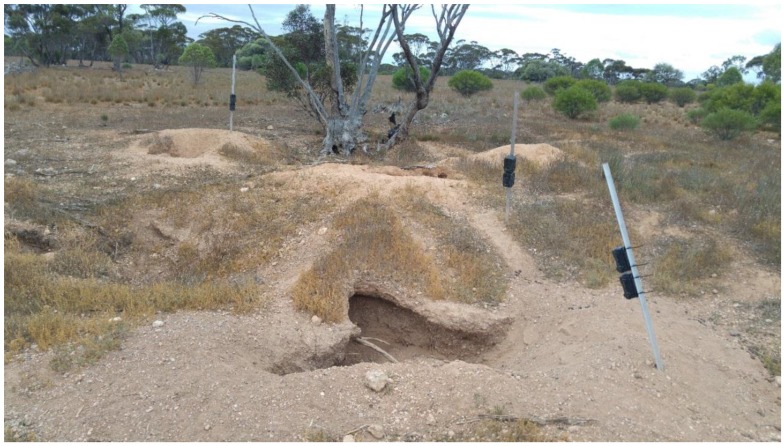
Camera set-up at Site #5, Moorunde Wildlife Reserve. Each pole has two cameras—one still and one video (six cameras total at each site), with all cameras aimed at the burrow crater in the foreground. The close-up cameras are on the right side of the image, the medium-range are in the centre, and the long-range cameras are on the left.

Phase 1 was conducted in the South Australian Murraylands region at two locations—the Moorunde Wildlife Reserve (34.41° S, 139.49° E) and the Brookfield Conservation Park (34.36° S, 139.49° E)—from 17 October to 12 November, and 15 November to 10 December 2017 respectively. Five active wombat burrows were selected at each location (ten active burrows overall) (Table 1). Burrows were considered active if there were signs of recent diggings (the past few days), fresh scats or footprints, or visual or audible signs of a wombat in the burrow. At each burrow, six Shenzhen Byhann BH-H801WP trail cameras, each with 32 Gb of data storage capacity, were mounted on three poles (two cameras—one still and one video—per pole), for a total of 30 cameras (15 video and 15 still) at each location. The cameras were attached to the pole one above the other (order determined randomly), with the lower camera approximately 60 cm from the ground. One pole (close-up) was installed on the spoil mound ~2 m from, and angled to face directly into, the burrow entrance. A second pole (medium-range) was located 3–5 m to the side of the burrow, and a third (long-range) was located 8–10 m from the burrow to obtain an overall view of the warren area (Figure 2). In order to minimise interference to the animals’ behaviour, we avoided positioning the camera poles on any burrow entry/exit paths or inter/intra-warren trails.

The still camera was configured to capture three x five-megapixel images (2592 × 1944 pixels) each time the camera was triggered, with a pause of five seconds between image sequences. These settings were the maximum number of images per sequence and the shortest pause time available on the camera. The video camera was configured to take a one-minute 720 P video (1280 × 720 pixels) each time the camera was triggered, with a pause of five seconds between subsequent recordings. The manufacturer’s claimed triggering speed of the cameras was 0.6 s.

### 2.2. Data Analysis

The first two days of imagery from each location were discarded to reduce any potential bias in the wombats’ behaviour which may have been caused by disturbance during the installation of the cameras [13]. As wombats can remain underground for long periods to avoid detection, we required a minimum of 10 days of recorded images from at least one camera for a site to be considered for detailed analysis [14]. The captured imagery was analysed by separating the images and videos into those that contained wombats, those that were triggered by animals other than wombats, and those for which no obvious triggering mechanism was apparent (‘false positives’).

The time and date of all the images and videos which contained wombats and all false positive videos were then noted and collated. We then examined all the wombat images and videos to determine whether we could positively identify a wombat entering or exiting a burrow, or whether they showed other activity unrelated to burrow entry/exit. We also examined the imagery to determine whether the burrow was being occupied by more than one wombat simultaneously. Only adult wombats were counted; joeys were excluded from our calculations. As wombats are nocturnal, we would generally expect them to exit the burrow in the late afternoon/evening, potentially visit other burrows during their active period or re-enter their original burrow across the night and then finally re-enter a burrow in the early morning where they would shelter during the day. We therefore considered a burrow to be occupied if there was evidence of a wombat entering the burrow in the morning and remaining inside during the middle of the day. The results were collated by camera pairings (close-up versus medium-range versus long-range) and by video versus still cameras.

All data was collated on a Microsoft Excel spreadsheet (2013 version 15), and statistical tests were conducted using the ‘Data Analysis’ tools add-in. The ‘Burrow Occupation Rate’ for each burrow was calculated by the dividing the number of days each burrow was occupied by one or more wombats by the number of valid observation days for that burrow. The ‘Wombat Occupancy Rate’ was calculated in the same manner as the ‘Burrow Occupancy Rate’, but was adjusted to take into account days when the burrow was occupied by more than one wombat simultaneously. The overall occupancy rate for each survey location was calculated by pooling the data for all burrows at that location, to account for any variations in the number of observation days per burrow. The ‘false positives’ for all video cameras were pooled and collated by time of day when they occurred.

Data from the camera positions (close vs. medium vs. long-range) and camera types (video vs. still) were compared using a non-parametric test for related samples. The data was initially checked for normality (skewness and kurtosis), then analysed using the Wilcoxon signed-rank test for two related samples.

### 2.3. Phase 2: Eyre Peninsula

Phase 2 was conducted at the Dakalanta Conservation Park (33.46° S, 135.34° E) on the Eyre Peninsula of South Australia, from 10 May to 7 June 2018. Ten active wombat burrows were selected for monitoring (Table 1).

Only the close and medium range video cameras were used; the long-range cameras and the still cameras were dispensed with. The camera settings were changed from those used during phase one to reduce the resolution of the video from 720 P to 320 × 240 pixels. The cameras were also set to revert to standby mode between 10:30 and 14:30 h each day, with no video recorded during that time. A single still camera was also mounted on the same pole as the ‘medium’ video camera, and was set to capture imagery between 10:00–15:00 each day (i.e., during the time that the video cameras were in standby mode). This camera was used solely to confirm that no animals had left the warrens whilst the video cameras were in standby mode. The same analysis methodology that was used during phase one was also used during this phase, except that the analysis of false positives was not conducted at this site.

## 3. Results

### 3.1. Phase 1

Although we installed cameras at five burrow sites in both locations in the Murraylands (ten sites overall), all six cameras at one site (Brookfield site #5) did not operate as they were not switched on correctly (set to ‘test’ instead of ‘on’). Of the nine remaining sites (54 cameras), ten video cameras stopped recording before the end of the survey period due to the data storage capacity (32 Gb) being reached (Moorunde site #1 long—9 days; site #3 close—9 days; site #3 long—5 days; site #4 close—14 days; site #4 long—19 days; site #5 close—13 days; site #5 medium—16 days; Brookfield site #2 close—14 days; Brookfield site #3 close—22 days; Brookfield site #4 close—21 days).

In total over 15,000 still images and videos were captured for analysis, of which 509 videos and 948 still images were of wombat activity. There were 1948 false positive videos which did not appear to have any obvious triggering event within the field of view.

**Figure 3:**
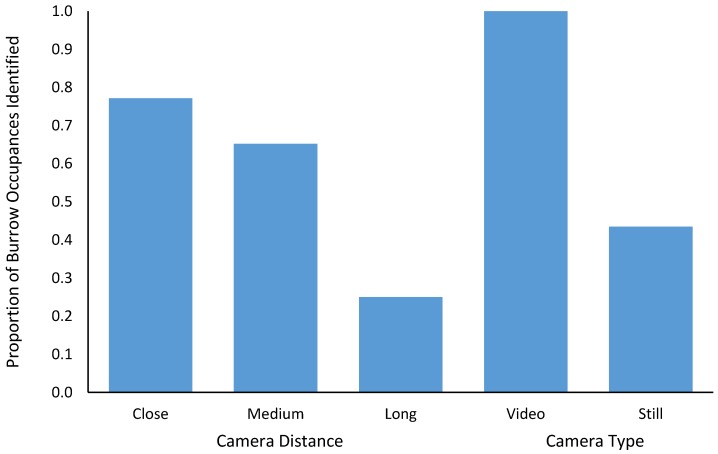
The proportion of burrow occupancies (entry into or exit from a burrow which provided positive evidence of a wombat occupying the burrow during the day) which we could identify from imagery recorded on different camera combinations.

We were able to positively identify 92 instances of a burrow being occupied from the video or still imagery. All 92 of these burrow occupancies could be confirmed on the video imagery, whereas we could only positively confirm 40 (43%) from the still imagery (Wilcoxon signed rank test Z = −2.032, *p* = 0.042). The distance at which the cameras were installed from the burrow entrance also affected our ability to identify whether a burrow was occupied. The cameras situated close to the burrow entrance successfully identified that a burrow was occupied on 71/92 occasions (77%) versus 60/92 for the medium-range cameras (65%) and 23/92 (25%) for the long-range cameras. A Wilcoxon signed-rank test showed that there was no significant difference between the close-up and the medium-range cameras (Z = −0.339, *p* = 0.735), but there was a significant reduction in our ability to positively confirm burrow occupancy from images derived from the long-range cameras (Z = −2.371, *p* = 0.018) (Figure 3).

**Figure 4:**
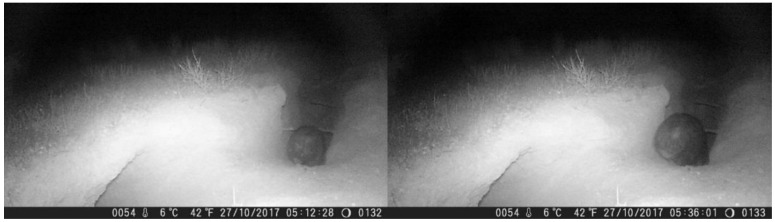
Video captures from the close-up video camera at Moorunde site #4 showing an example of simultaneous burrow sharing by two wombats. In the left image (captured from video # 0132), a wombat enters the burrow at 05:12. Twenty-four minutes later at 05:36, recorded on the subsequent video (# 0133), a second wombat enters the same burrow.

Eight out of nine of the burrows for which we had data were occupied by a wombat or wombats at some time during the survey period. The occupancy rate varied from 0% (burrow assessed as active but not occupied at any time during the survey period) to 88% (burrow occupied for 22/25 days). Of the eight burrows which were occupied, two were occupied for less than 25% of the time (12% and 24%), two were occupied for 25–50% of the time (32% and 40%), three were occupied for 50–75% of the time (56, 56, 60%), and one was occupied for more than 75% of the time (88%). Two burrows (Moorunde sites #4 and #5), were occupied by two wombats simultaneously for a total of two and three days respectively (Figure 4) (Table 2).

The burrow occupancy rate—the proportion of burrows which were occupied at any one time—during this phase of the study was 0.41 (SD = 0.26). The wombat occupancy rate, and hence the index of wombats/active burrow for the Murraylands, was 0.43 (SD = 0.29).

**Figure 5:**
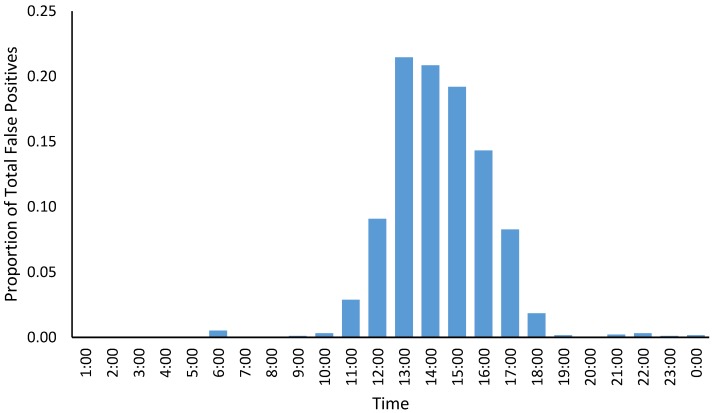
Proportion of false positive videos (videos for which there was no obvious triggering event and for which there was no animal visible in the field of view) by time of day. Over 85% of all false positive videos occurred in the afternoon between 12:00 and 18:00, with 95% occurring between 11:00 and 18:00.

Of the 1948 false positive videos, 1464 were from Moorunde and 484 were from Brookfield. 1674 of the false positive videos (86%) occurred in the afternoon between 12:00 and 18:00, with only 27 (2%) occurring at night between 20:00 and 06:00 (Figure 5).

### 3.2. Phase 2

Although we installed cameras on ten burrows, the cameras on one burrow (site #4) were dislodged by a herd of deer after six days of operation, leaving only four days of valid observations. This site was therefore discarded from further analysis. A second set of cameras (site #10) was also dislodged by the deer after 14 days of operation, but as this provided 12 days of data this site was included in our analysis. Seven of the remaining eight sites provided a full 25 days of data for analysis, with the data storage capacity of the cameras at the remaining site (site #2) being filled after 20 days (18 days of data for analysis).

Eight out of nine burrows for which we had valid data were occupied by a wombat or wombats at some time during the survey period. The burrow usage rate—the proportion of days when the burrow was in use by one or more wombats—varied from 0% (site #1: burrow assessed as active but not occupied at any time during the survey period) to 100% (site #2: burrow occupied every day during the survey period). Of the eight burrows which were occupied, two were occupied for less than 25% of the time (both at 8%), four were occupied for 25–50% of the time (25%, 28%, 40%, 40%), none were occupied for 50–75% of the time, and two were occupied for more than 75% of the time (76% and 100%) (Table 2). Two burrows (sites #2 and #3), were occupied simultaneously by more than one wombat for twelve and three days respectively.

The mean burrow occupancy rate—the proportion of burrows which were occupied at any one time—during this phase of the study was 0.35 (SD = 0.33). The overall wombat occupancy rate—the mean number of wombats/active burrow—for the Eyre Peninsula, was 0.42 (SD = 0.54).

## 4. Discussion

This study informs two important areas of wombat population research; the reliability of cameras as tools for monitoring wombat activity, and the calculation of an index of the number of wombats/active burrow for use in population abundance estimates. Our results provide evidence for the efficacy of video as a tool for determining warren occupancy rates, albeit with some limitations. They also provide lessons in regard to understanding how the cameras work, and ensuring that they are properly sited and the correct settings are used.

Comparisons between the video and still imagery collected during phase one of this study indicated that there was a clear difference in our ability to positively identify wombat burrow occupancy from video versus the still imagery. The identification of burrow occupancy from video was relatively simple, whilst the approach used in previous studies of identifying individual wombats from still imagery can be difficult and time consuming, with identification relying on characteristics (nose hair patterns, scars, ear notches, etc.) that are not always visible in the imagery. Further, because wombats do not just reside in one warren, but rather use up to ten different warrens throughout their home range [7], accounting for wombats which might be ‘just visiting’ a warren, as opposed to being a resident of that warren, is problematic. We have cases where we have counted over 30 different wombats using 14 burrows over a period of one month; which equates > 2 wombats/active burrow. Consequently, counting wombats would clearly require more detailed statistical analysis than just assessing burrow occupancy in order to produce accurate abundance estimates.

The failure of the long-range cameras to contribute much useful information during phase one of the study (25% of burrow occupancies identified) highlights some of the limitations with using cameras for this type of research [15]. There were numerous occasions when data was captured on the close-up and medium-range cameras, but nothing was recorded on the long-range cameras. This suggests that the distance (~10 m) may have been beyond the cameras’ range, despite the manufacturers claiming a detection range of up to 20 m. We suggest that this is probably only applicable to larger animals, as we did not observe wombats at anything approaching that distance.

The Passive Infra-Red (PIR) sensors used on these cameras detect the surface temperature of objects in the field of view, and trigger the camera when a change in temperature is detected in part or all of the sensor [16]. This occurs when an object with a different surface temperature to its surrounds changes its aspect in relation to the sensor (i.e., moves across the field of view). However, the cameras sometimes trigger when there is no obvious target within their field-of-view. We noted that this phenomenon was particularly prevalent during the daytime, with 86% of all false positive detections that we recorded occurring in the afternoon, and less than 2% occurring at night. Although we did not analyse the false positive rate for phase two, given that we set the cameras to revert to standby mode between 10:30 and 14:30 each day, we noticed that there were few false positive video recordings on any of these cameras, and of the few that did occur, almost all were between 14:30 and 18:00.

Whilst previous studies have suggested that these false positives may be triggered by the wind moving vegetation within the field-of-view of the camera [17], our results suggest that other factors may have a greater effect. For example, we noticed that while false positives did occur frequently on windy days, they also occurred at a similar rate on still days when neither the camera nor branches were in motion. The increasing number of false positives which occurred as the day progressed and the lower rate of false positives recorded during the night suggests that it may be solar radiation causing the uneven heating of the ground in front of the camera, or heating of the camera body itself, which causes the cameras to trigger. We recommend that further research and pre-survey trials be conducted before undertaking future wildlife studies of this nature, to verify the causes and rate of false positives for the particular camera model being used.

The high rate of false positives, coupled with the data requirements of high-definition (720 P) video, meant that we used up the data storage capacity (32 Gb) of some of the video cameras before the end of the planned survey period during phase one of the study. As a result, we reduced the resolution of the videos to the minimum size possible on the cameras (320 × 480 P) to reduce the data storage requirements for phase two of the study. While this largely solved the problem of data capacity, the high volume of wombat traffic at one site (Dakalanta site #2) still resulted in the data capacity of the cameras at that site being used up after 20 days. This highlights the need to carefully evaluate the potential volume of data which may be captured by a camera and the data storage capacity available, and to adjust the camera settings, survey periods and logistics of any study to ensure that data is not missed as a result of inadequate data storage capacity.

**Figure 6:**
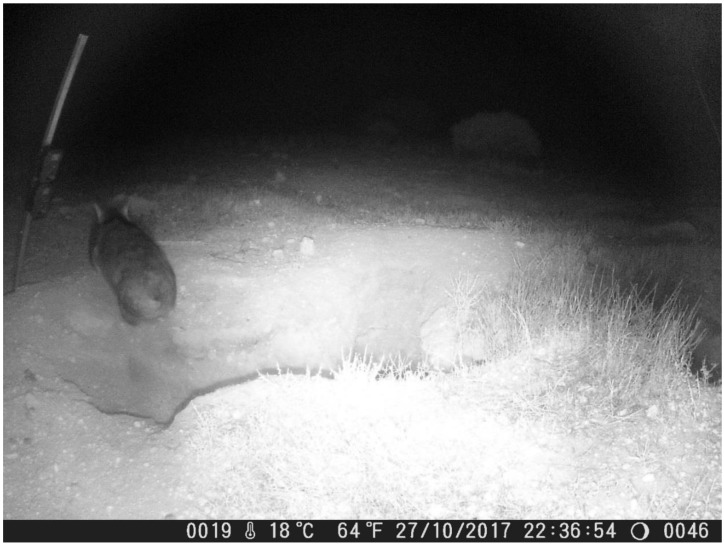
While this image of a wombat emerging from its burrow was captured on both the medium-range video and still cameras (medium-range still camera image shown)—which were oriented side-on to the burrow entrance—it was not recorded on either of the short-range cameras (the pole in the image) which were facing directly at the burrow entrance. This highlights the potential for single camera studies to fail to capture imagery of some animals even if they are clearly within their field-of-view, and for the need to consider the orientation of cameras in relation to expected target motion.

In regard to the siting of the cameras around warrens, in the case of a wombat emerging from its burrow there would be only a limited aspect change in relation to the camera oriented directly into the burrow (i.e., the close-up camera). The surface temperature of the wombat’s body is also likely to be similar to the ambient temperature of the burrow, especially when the wombat is covered in dirt and the weather is warm. This suggests that fossorial species such as wombats may represent a poor target for PIR detection. There was evidence for this in our observations of burrow entry/exit. Although we generally observed the entire burrow entry process from the video (the wombat approaching, descending into the burrow crater and entering the burrow), in the case of burrow exits the camera usually did not trigger until the wombat had completely emerged from the burrow and was climbing out of the crater (i.e., we rarely observed the wombat inside the mouth of the burrow). We also noted cases where imagery of a wombat exiting the burrow would be captured on the medium-range camera, which was oriented side-on to the burrow entrance, but no imagery was captured on the close-up camera (Figure 6). This phenomenon of cameras failing to detect animals which were within their field of view has been previously described, with up to 68% of verified animal activity being missed in some circumstances [17].

Whether the failure of some of our cameras to detect wombat activity which was occurring within their field of view—and which was captured on other cameras with a different orientation to the target—is an inherent limitation of wildlife trail cameras, or whether it is a limitation of these type of cheaper, generic cameras which are entering the market is difficult to tell from our data. In either case, our findings provide strong support for the importance of understanding the capabilities and limitations of motion-activated infra-red cameras in general, and of the model of camera being used, prior to their use in studies of this type. It also underscores the importance of the correct placement and alignment of the camera in relation to the target and to minimise the potential for false triggers to occur [15]. Unfortunately, it may not be possible to overcome all the potential limitations with a single camera no matter its quality or how well it is sited, and hence multiple cameras located in different positions may be necessary in some situations. If this is the case, it raises questions about the findings from previous studies where only one camera was used to detect animal presence/activity, and whether some data might have been missed as a result. We recommend further research in this area.

In regard to our calculation of the number of wombats/active burrow, our figure of 0.43 for the Murraylands is virtually identical to previous figures calculated for the region of 0.43 [6], 0.4389 [2,13] and 0.40 [18]. The figure of 0.42 for the Eyre Peninsula is the first index which has been calculated for that area. Both figures are lower than 0.60 calculated for the Far West Coast [5] and 0.50 which was used for the only species-wide abundance estimates undertaken to date [19].

Whilst other studies suggest that a range of indices are required for different conditions such as soil type, especially for small-scale population estimates in areas with a large number of calcrete warrens [4,20], the remarkable similarity between the figures we calculated for the Murraylands and Eyre Peninsula suggests that, at the broad scale at least, an index of ~0.43 might be a robust figure to calculate wombat abundance. Nonetheless, as this issue is fundamental to our understanding of wombat abundance at both the local and broad scales, we would recommend on-going research to validate these findings, especially in regions other than those previously surveyed and/or under a range of environmental conditions such as drought [13,21].

Our results also highlight the difficulties with accurately assessing whether a burrow is active, an issue which has been previously identified in multiple studies [6,13,22]. Two survey sites, site #1 at Moorunde and site #1 at Dakalanta were both assessed as active, based on recent diggings and fresh wombat scats around the burrow entrances. However, whilst we observed wombat activity in the vicinity of the burrow entrance at both sites throughout several nights during the survey period, as well as activity by other species including kangaroos and feral goats, there was no evidence on the imagery of a wombat entering or exiting either burrows at any time during the survey. It appears that activity by both visiting wombats and non-target species around the entrance to the burrows disturbed the soil, and together with the deposition of scats, resulted in the misidentification of the burrows’ status. We also noted the phenomenon at other survey sites. For example, although the burrow occupation rate for Dakalanta site #5 was only 0.08 (burrow occupied for 2 out of 25 survey days), the signs—digging, footprints, fresh scats—around the burrow entrance suggested a high rate of activity. This was supported by the video evidence, which showed wombat activity in the vicinity of the burrow, including digging around the burrow entrance, on all but two nights (23/25 nights) of the survey period.

Whether these inaccuracies in the subjective assessment of a burrow’s active status constitutes a problem depends upon the context. Certainly, at the local scale, if research effort is spent on a burrow which is not active, then that effort may be wasted. Conversely, as the calculated indices of the number of wombats/active burrow are all based on subjective assessments which also includes the misidentification of the status of some burrows [2,5,6,13], we do not consider this to be a significant problem for broad area population studies. Nonetheless, consideration should be given to using small scale studies as models to develop indices prior to any broad-scale population survey, and to ensure that subjective assessments by multiple researchers are standardised to reduce potential errors in the abundance calculations.

**Figure 7:**
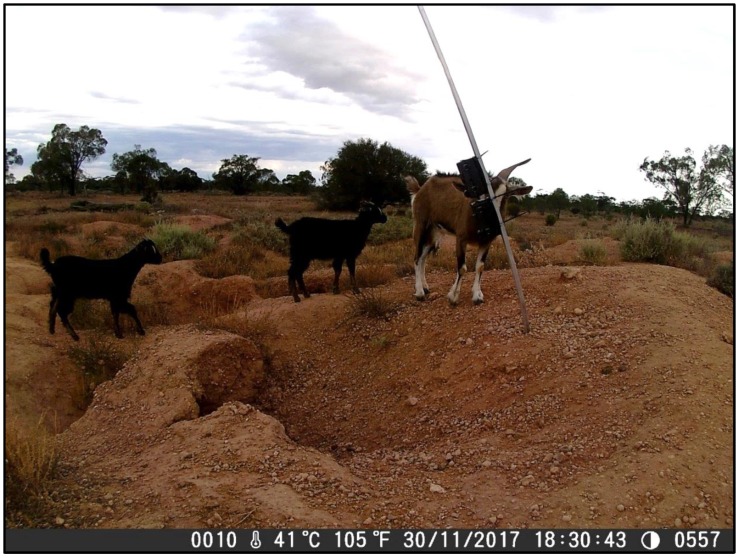
Feral goats examining one of our camera poles in Brookfield Conservation Park (site #1). The close-up cameras are visible in the image, which was captured on the medium-range camera. Note how the temperature at 18:30 is still 41 °C. It is likely that a combination of high temperatures and disturbance by the goats discouraged the wombat from emerging from its burrow during the nights of 29 and 30 November.

We also noted days where no wombat activity was captured on the cameras—and hence, we did not observe a wombat entering or exiting the burrow—but we assessed that the burrow was occupied. This can be explained by the observation of a wombat entering the burrow but not emerging until several days later. We noted three instances of a wombat remaining in its burrow for more than one day, with the longest period being three and a half days (wombat entered at 05:07 on 29 November and did not emerge until 21:29 on 2 December). A second wombat also did not emerge from a different burrow for two days during the same period. This was most likely related to the weather, with our cameras recording maximum temperatures in excess of 40 °C on both 29 and 30 November 2017. A herd of feral goats was also observed grazing on the surface of the warren on the evening of the 30 November (Figure 7). This avoidance behaviour by wombats of high temperatures and outside disturbances has been previously described by multiple sources [13,23,24,25,26]. It also highlights the merits of non-invasive approaches which reduce the potential to cause behavioural changes, and of surveys which investigate burrow occupancy rather than wombat activity, which is likely to vary seasonally and in response to environmental conditions.

In regard to burrow sharing by more than one wombat, while this is not thought to be common, it does occur, especially in areas dominated by layers of sheet calcrete limestone [27] where access to the subterranean shelter may be limited to breaks or fractures sites in the limestone layer. Previous studies have also suggested that these warrens can have more complex underground structures; with large open areas between the calcrete layers and several chambers which are accessed from a single burrow entrance [28]. We noted four sites (out of 18 total survey sites) where a single burrow entrance was used simultaneously by more than one wombat; Moorunde sites #4 and #5, and Dakalanta sites #2 and #3. For three out of four of these sites, burrow sharing occurred on only a few days out of the survey period. At Dakalanta site #2 burrow sharing occurred across two-thirds of the survey period (12 out of 18 days), with three wombats being observed to share the burrow for one of those days. As a consequence, we would recommend that further research be undertaken to ascertain burrow sharing behaviour and kinship relationships among adult wombats [29,30].

With studies of this nature there is always the possibility that the cameras did not capture all the wombat activity, Although the number of cameras that we used reduces the likelihood of this occurring, we cannot discount the possibility that we may have missed one or more burrow occupancies. As a consequence, our indices of 0.43 for the Murraylands and 0.42 for the Eyre Peninsula should be considered to be minimum figures, and further research should be undertaken to verify these findings.

## 5. Conclusions

The use of motion-activated wildlife cameras has become more prevalent over the past 20 years, but there is still a great deal that researchers can and should learn about their capabilities and limitations, if they are to gain the maximum benefit from their use. Some of our results surprised us, especially when we observed wombat activity on one camera but saw nothing on another camera that had the same focal area covered. We, therefore recommend that pre-survey trials be undertaken before using cameras in the field to ensure their capabilities and limitations are fully understood, and camera settings, siting and alignment to the target are optimised. The use of multiple cameras with different positions and alignments should also be considered to overcome any potential limitations inherent in a single camera design. For surveys involving fossorial species we recommend that cameras be oriented at an angle to, rather looking directly into, the burrow.

We found that it was much easier to determine wombat behaviour and burrow occupancy rates from video than still imagery, and we recommend the greater use of video for studies of this nature.

The index of wombats/active burrow that we calculated (0.43) for the Murraylands was identical to previous studies for the region, whilst the index calculated for the Eyre Peninsula (0.42) was virtually identical. This suggests that these figures are probably robust enough for most broad-scale wombat population studies. However, we recommend that on-going studies of this nature be undertaken to refine our understanding of southern hairy-nosed wombat burrow occupation rates at different spatial scales and under different environmental conditions.

## Figures and Tables

**Table 1:** Survey burrows.

Site #		1	2	3	4	5	6	7	8	9	10
Moorunde	Warren Diameter (m)	10	13	11	15	12					
	No. of Active Burrows	1	2	3	4	5					
	No. of Burrows	3	5	6	8	6					
Brookfield	Warren Diameter (m)	22 ^1^	22 ^1^	22 ^1^	17 ^2^	17 ^2^					
	No. of Active Burrows	4	4	4	3	3					
	No. of Burrows	12	12	12	10	10					
Dakalanta	Warren Diameter (m)	11	13	6	16	12	9	14	19	5	7
	No. of Active Burrows	2	2	1	5	2	2	3	4	1	1
	No. of Burrows	4	3	1	6	5	3	8	6	1	1

^1,2^ While the Moorunde and Dakalanta sites were ‘stand-alone’, with only one burrow being surveyed and one camera group on each warren, the Brookfield sites (^1^ and ^2^) had more than one survey burrow per warren.

**Table 2:** Proportion of days during the survey period when the surveyed burrow was occupied by one or more wombats, and the wombat occupancy rate. Four burrows (Moorunde sites #4 and 5 and Dakalanta sites #2 and 3) were occupied by more than one wombat on two, three, twelve and three days respectively.

Site #		1	2	3	4	5	6	7	8	9	10	Mean
Moorunde	Burrow Occupied	0.00	0.56	0.12	0.60	0.88						0.43
	Wombat Occupancy	0.00	0.56	0.12	0.68	1.00						0.47
Brookfield	Burrow Occupied	0.40	0.24	0.32	0.56	-						0.38
	Wombat Occupancy	0.40	0.24	0.32	0.56	-						0.38
Dakalanta	Burrow Occupied	0.00	1.00	0.76	-	0.08	0.40	0.08	0.40	0.28	0.25	0.35
	Wombat Occupancy	0.00	1.72	0.88	-	0.08	0.40	0.08	0.40	0.28	0.25	0.42

## Supplementary Materials

The imagery and videos captured for this study have been deposited in https://figshare.com/account/home#/collections/4241273.
